# Evaluation of adjunctive mycophenolate for large vessel giant cell arteritis

**DOI:** 10.1093/rap/rkaa069

**Published:** 2020-12-19

**Authors:** Maira Karabayas, Paula Dospinescu, Nick Fluck, Dana Kidder, Gillian Fordyce, Rosemary J Hollick, Cosimo De Bari, Neil Basu

**Affiliations:** 1 Aberdeen Centre for Arthritis & Musculoskeletal Health, University of Aberdeen; 2 Rheumatology Service, NHS Grampian; 3 Renal Unit, Aberdeen Royal Infirmary, NHS Grampian, Aberdeen; 4 Institute of Infection, Immunity & Inflammation, University of Glasgow, Glasgow, UK

**Keywords:** Large vessel vasculitis, giant cell arteritis, mycophenolate, steroid sparing, relapse rate

## Abstract

**Objectives:**

GCA patients with large vessel involvement (LV-GCA) experience greater CS requirements and higher relapse rates compared with classical cranial GCA. Despite the distinct disease course, interventions in LV-GCA have yet to be investigated specifically. This study aimed to evaluate the CS-sparing effect and tolerability of first-line mycophenolate in LV-GCA.

**Methods:**

A retrospective cohort study was conducted in patients with LV-GCA identified from a regional clinical database between 2005 and 2019. All cases were prescribed mycophenolate derivatives (MYC; MMF or mycophenolic acid) at diagnosis and were followed up for ≥2 years. The primary outcome was the cumulative CS dose at 1 year. Secondary outcomes included MYC tolerance, relapse rates and CRP levels at 1 and 2 years.

**Results:**

A total of 37 patients (65% female; mean age 69.4 years, SD 7.9 years) were identified. All cases demonstrated large vessel involvement via CT/PET (*n* = 34), CT angiography (*n* = 5) or magnetic resonance angiography (*n* = 2). After 2 years, 31 patients remained on MYC, whereas 6 had switched to MTX or tocilizumab owing to significant disease relapse. The mean (±SD) cumulative prednisolone dose at 1 year was 4960 (±1621) mg. Relapse rates at 1 and 2 years were 16.2 and 27%, respectively, and CRP levels at 1 and 2 years were 4 [interquartile range (IQR) 4–6] and 4 (IQR 4–4) mg/l, respectively.

**Conclusion:**

To our knowledge, this is the first attempt to assess the effectiveness of any specific agent in LV-GCA. MYC might be both effective in reducing CS exposure and well tolerated in this subpopulation. A future randomized controlled trial is warranted.

**Key messages**

- It is feasible to evaluate therapeutics specifically in GCA with large vessel involvement.
- Mycophenolate derivatives appear to reduce CS exposure in GCA with large vessel involvement.

## Introduction

GCA is the commonest form of adult systemic vasculitis. In Northern Europe, incidence rates range between 13 and 22 per 100 000 among individuals >50 years of age [1, 2]. Although classically considered a disease of the cranial arteries, greater access to vascular imaging has revealed prevailing involvement of larger blood vessels, such as the aorta, in 29–83% of patients [3]. Such heterogeneity is associated with differences in outcomes. Prominent large vessel involvement in GCA (LV-GCA) confers a greater CS exposure, risk of relapse and risk of aortic aneurysm formation, whereas visual complications are less likely [3–6]. Furthermore, although studies of GCA immunobiology have historically been limited to the temporal artery, Brack *et al.* [7] previously reported immune transcriptomic variation across different-sized blood vessel walls in GCA (aorta *vs* temporal artery), including cytokine transcripts and HLA-DR allele expression. More recently, Graver *et al.* [8] provided evidence of predominant B-cell infiltrates in GCA diseased aortic tissue, which contrasts with the well-characterized CD4^+^ Th cell preponderance of GCA diseased temporal arteries [9]. Together, these data support the existence of distinct endotypes, aligned to vessel size, which, in all probability, will manifest different responses to different treatments. As a minimum, it is therefore compelling to test the efficacy and safety of interventions for GCA separately according to cranial or large vessel predominance. Remarkably, no study has been designed specifically to evaluate an intervention exclusively in LV-GCA; existing studies are limited to either isolated cranial disease or a mixture of the two variants [10].

CSs are a major established source of GCA morbidity [11]. In the absence of evidence, and in recognition of the excess of CS use in LV-GCA, our regional centre has routinely prescribed adjunctive mycophenolate derivatives (MYC) for LV-GCA at diagnosis in order to minimize cumulative CS exposure.

MYC inhibits type I and II inosine monophosphate dehydrogenase, which in turn prevents synthesis of guanine monophosphate. Unlike other cell types, B and T lymphocytes rely upon guanosine nucleotides; therefore, MYC suppresses their proliferation [12]. Despite the lack of published evidence, the putative role of these cell sets in the pathogenesis of GCA and the proven efficacy of MYC in other forms of systemic vasculitis [13] have led to its use in clinical practice for GCA. It is, however, not recommended in national guidelines owing to the lack of published evidence [14]. Indeed, only a single study has been reported. Although positive, it was a small anecdotal case series (*n* = 3), limited to cranial GCA [15].

The aim of this study was to be the first to evaluate the CS-sparing effectiveness and tolerance of adjunctive MYC in GCA patients with large vessel involvement at diagnosis.

## Methods

We conducted a retrospective cohort study. Cases were patients with a physician diagnosis of LV-GCA and evidence of large vessel vasculitis on imaging. All cases prescribed adjunctive MYC at diagnosis with a disease duration of ≥2 years were included. Adjuvant MMF is considered the first-line standard of care in our region, prescribed to a target dose of 1 g twice a day. For those who experience persistent gastrointestinal side effects, this is often switched to mycophenolic acid to a target dose of 720 mg twice daily. Patients were identified from the National Health Service (NHS) Grampian vasculitis service database, a regional clinic in Scotland, which serves a population of ∼585 700 [16]. The clinical database provided details of all patients between June 2005 and September 2019. Diagnosis was substantiated after review of medical records.

Baseline characteristics and outcomes were extracted from electronic case records. Baseline characteristics included demographics, clinical features and diagnostic investigations. The primary outcome was 1 year cumulative CS dose. Secondary outcomes included MYC intolerance, at 1 and 2 years, relapse rates, defined as a recurrence in LV-GCA symptoms requiring an enhancement of immunosuppression (including CSs), and CRP at 1 and 2 years.

Simple descriptive statistics were computed and are presented as the mean (±SD) or median [interquartile range (IQR)] according to the distribution of each variable. No ethics approval was required. The study was a clinical service evaluation and complied with local institutional governance (Project ID 4959).

## Results

Of the *n* = 37 LV-GCA cases who met inclusion criteria, *n* = 24 (65%) were female, with a mean (SD) age at diagnosis of 69.4 (7.9) years, and *n* = 31 (83.8%) fulfilled the 2012 Chapel Hill Consensus conference definition for GCA. Constitutional upset was the commonest presenting complaint (*n* = 32, 86.5%), followed by polymyalgia (*n* = 26, 70.3%) and headache (*n* = 23, 62.1%), and *n* = 9 (24.3%) reported visual disturbance. All patients recorded a raised systemic inflammatory response, as measured by CRP/ESR. In terms of imaging, evidence for large vessel vasculitis was established by PET/CT in *n* = 34; diagnosis of the remainder was supported by magnetic resonance angiography (*n* = 2) and CT angiography (*n* = 5) (Table 1).

**Table 1:** Demographics and baseline disease characteristics

Parameter	Total *n* = 37
Age, years, mean (±SD)	69.4 (±7.9)
Female, *n* (%)	24 (65)
Fulfil CHCC 2012, *n* (%)	31 (83.8)
CRP, mg/l, median (IQR)	71 (38.3–139)
ESR (*n* = 24), mm/h, mean (±SD)	76 (±24.7)
TAB (*n* = 8), positive, *n* (%)	2 (25)
PET/CT (*n* = 36), positive, *n* (%)	34 (94.4)
CTA (*n* = 5), positive, *n* (%)	5 (100)
MRA (*n* = 2), positive, *n* (%)	2 (100)

CHCC: Chapel Hill Consensus Conference; CTA: CT angiography; IQR: interquartile range; MRA: magnetic resonance angiography; TAB, temporal artery biopsy.

In total, *n* = 31 tolerated and remained on MYC after 2 years (*n* = 27 MMF and *n* = 4 mycophenolic acid), whereas *n* = 6 switched to MTX or tociluzimab owing to significant disease relapse (defined as recurrence of symptoms and/or evidence of disease activity or structural progression on imaging). The median (IQR) cumulative prednisolone dose, 1 year after diagnosis, was 4882.5 (3887–5646.5) mg. Relapse rates after 1 and 2 years were 16.2 and 27%, respectively. CRP levels at 1 and 2 years were 4 (IQR 4) and 4 (4–4) mg/l, respectively, and were not significantly different (*p* = 0.53).

## Discussion

In this first study to evaluate a therapeutic agent specifically in LV-GCA, adjunctive MYC therapy at diagnosis was associated with a cumulative prednisolone exposure in the first year of <5 g. Relapse rates remained relatively low at 1 and 2 years, and overall, the drug was well tolerated by patients.

These results are encouraging when compared with historical LV-GCA outcomes. Data from the Mayo Clinic estimated a mean cumulative CS exposure in the first year that was more than twice that observed in the present study (11.1 *vs* 5.0 g) [3]. Moreover, in the same study, a relapse rate of 60% was observed in the first year among *n* = 103 LV-GCA patients (compared with 16.2% reported herein). Of these, 32 received at least one concomitant immunosuppressive within a year of diagnosis and only *n* = 5 received MYC during the median 3.6 year follow-up period. More recently, de Boysson *et al.* [17] reported relapse rates of 39% amongst 248 patients with imaging-proven LV-GCA, and only 28% received additional immunosuppression.

In ANCA-associated vasculitis, MMF was also found to be well tolerated [13]; however, LV-GCA patients are generally older and therefore relatively vulnerable to toxicity. A UK study, published only as a conference abstract, examined the use of MYC in a large vessel vasculitis cohort (*n* = 35), including *n* = 5 GCA cases. The drug was tolerated to a similar extent, with only 9% discontinuing therapy owing to side effects and almost all (97%) experiencing a CS-sparing effect. Unfortunately, relapse rates or cumulative CS doses were not reported [18]. The comparable extant literature is otherwise limited, but a French cohort identified the use of immunosuppressants generally to be associated with an improved LV-GCA outcome [4]. Indeed, international guidelines already recommend adjunctive immunosuppression (tociluzimab or MTX) in selected GCA patients [19]. Although this recommendation is informed by high-quality randomized controlled data, the selection criteria of the source trials included both cranial and LV-GCA, and separate analyses of these subgroups have not been reported. It is therefore not possible to make direct comparisons of these outcomes, given the excess risks of relapse and CS exposure in LV-GCA compared with cranial GCA. In fact, the cumulative CS doses observed in studies such as GiACTA are relatively lower, even in the placebo groups (<4 *vs* 5 g), further supporting the rationale that these different disease variants manifest distinct disease courses and treatment responses [20]. However, even the combined data indicate that significant numbers of all GCA patients do not respond to either tociluzimab [20] or MTX [21]; therefore, alternative therapeutic options are necessary. Moreover, considering the apparent immunobiological differences between the cranial and LV variants, it is conceivable that treatment responses will vary further.

This study should be considered in the context of several limitations. Firstly, the study is retrospective and used electronic records for data extraction. In our region, however, electronic records are centralized, including laboratory, clinical and pathology records, and therefore, overall missing data were limited. Cumulative prednisolone doses were calculated for all patients retrospectively, which might have been associated intrinsically with a degree of error. Selection bias was minimized, because all patients in our region are managed by a single vasculitis service. That being said, these patients are often underdiagnosed owing to their non-specific presentations, and therefore, complete case capture was unlikely. Selection bias is further reduced by the protocolized approach of our service to the management of LV-GCA. Specifically, all patients during this time period started on MYC first line, apart from a single patient who was recruited to a randomized controlled trial. Data collection, however, is not protocolized, and therefore, some outcome data of interest (e.g. imaging and measures of quality of life) were not recorded systematically. Secondly, without controls these data can be contextualized only with the historical literature. A prospective randomized controlled trial is essential to determine the true effectiveness of this agent for LV-GCA; however, these current positive data do motivate such an undertaking in the future. Thirdly, definitions of GCA relapse vary between studies, and no consensus exists for specifically defining this principal outcome in LV-GCA. The main comparator study here used a slightly different definition of relapse [18]. Muratore *et al.* [3] required not only a recurrence in symptoms and change in immunosuppression to define relapse (in line with the present study), but also an increase in inflammatory markers. This more conservative characterization implies that the 43.8% difference in 1 year relapse rates observed between our studies is an underestimate, further strengthening the potential benefits of MYC.

## Conclusion

We have demonstrated the feasibility of conducting clinical evaluations in LV-GCA. We advocate further analyses of existing trial data sets to establish the presence, or not, of differential treatment effects according to vessel size and future powered studies that focus specifically on this apparent phenotypically and biologically distinct disease entity.

In summary, adjunctive MYC at diagnosis could be effective in reducing CS exposure and was well tolerated in LV-GCA. Given the clinical need for greater therapeutic choice, these data encourage further testing in a blinded, randomized clinical trial.

*Funding*: We are grateful to Versus Arthritis (grant 22088) and PMR/GCA Scotland for supporting our work.

*Disclosure statement*: N.B. has received non-promotional speaking fees from Roche, Abbvie, Vifor, Lilly and Pfizer and research funding from Pfizer, GSK, Vifor and Novartis. The other authors have declared no conflicts of interest.

## References

[1] Smeeth L, Cook C, Hall AJ. Incidence of diagnosed polymyalgia rheumatica and temporal arteritis in the United Kingdom, 1990–2001. Ann Rheum Dis 2006;65:1093–8. 16414971 10.1136/ard.2005.046912 PMC1798240

[2] Mohammad AJ, Nilsson JA, Jacobsson LT, Merkel PA, Turesson C. Incidence and mortality rates of biopsy-proven giant cell arteritis in southern Sweden. Ann Rheum Dis 2015;74:993–7. 24442881 10.1136/annrheumdis-2013-204652

[3] Muratore F, Kermani TA, Crowson CS et al Large-vessel giant cell arteritis: a cohort study. Rheumatology 2015;54:463–70. 25193809 10.1093/rheumatology/keu329 PMC4425829

[4] de Boysson H, Liozon E, Espitia O et al Different patterns and specific outcomes of large-vessel involvements in giant cell arteritis. J Autoimmun 2019;103:102283. 31130367 10.1016/j.jaut.2019.05.011

[5] Kermani TA, Warrington KJ, Crowson CS et al Large-vessel involvement in giant cell arteritis: a population-based cohort study of the incidence-trends and prognosis. Ann Rheum Dis 2013;72:1989–94. 23253927 10.1136/annrheumdis-2012-202408 PMC4112513

[6] Espitia O, Néel A, Leux C et al Giant cell arteritis with or without aortitis at diagnosis. A retrospective study of 22 patients with longterm followup. J Rheumatol 2012;39:2157–62. 22984271 10.3899/jrheum.120511

[7] Brack A, Martinez-Taboada V, Stanson A, Goronzy JJ, Weyand CM. Disease pattern in cranial and large-vessel giant cell arteritis. Arthritis Rheum 1999;42:311–7. 10025926 10.1002/1529-0131(199902)42:2<311::AID-ANR14>3.0.CO;2-F

[8] Graver JC, Boots AMH, Haacke EA et al Massive B-cell infiltration and organization into artery tertiary lymphoid organs in the aorta of large vessel giant cell arteritis. Front Immunol 2019;10:83. 30761147 10.3389/fimmu.2019.00083 PMC6361817

[9] Weyand CM, Goronzy JJ. Immune mechanisms in medium and large-vessel vasculitis. Nat Rev Rheumatol 2013;9:731–40. 24189842 10.1038/nrrheum.2013.161 PMC4277683

[10] Koster MJ, Matteson EL, Warrington KJ. Large-vessel giant cell arteritis: diagnosis, monitoring and management. Rheumatology 2018;57:ii32–ii42. 29982778 10.1093/rheumatology/kex424

[11] Buttgereit F, Matteson EL, Dejaco C, Dasgupta B. Prevention of glucocorticoid morbidity in giant cell arteritis. Rheumatology 2018;57:ii11–ii21. 29982779 10.1093/rheumatology/kex459

[12] Hiemstra TF, Jones RB, Jayne DR. Treatment of primary systemic vasculitis with the inosine monophosphate dehydrogenase inhibitor mycophenolic acid. Nephron Clin Pract 2010;116:c1–10. 20484933 10.1159/000314543

[13] Jones RB, Hiemstra TF, Ballarin J et al Mycophenolate mofetil versus cyclophosphamide for remission induction in ANCA-associated vasculitis: a randomised, non-inferiority trial. Ann Rheum Dis 2019;78:399–405. 30612116 10.1136/annrheumdis-2018-214245

[14] S M. BSR guideline on diagnosis and treatment of giant cell arteritis. British Society for Rheumatology, 2020 https://www.rheumatology.org.uk/Portals/0/Documents/Guidelines/GCA/Open_Consultation_Full_GCA_Guideline.pdf? ver=2019-07-02-152636-237 (26 January 2020, date last accessed).

[15] Sciascia S, Piras D, Baldovino S et al Mycophenolate mofetil as steroid-sparing treatment for elderly patients with giant cell arteritis: report of three cases. Aging Clin Exp Res 2012;24:273–7. 23114555 10.1007/BF03325257

[16] Government S. https://statistics.gov.scot/atlas/resource? uri=http%3A%2F%2Fstatistics.gov.scot%2Fid%2Fstatistical-geography%2FS08000020 (28 May 2020, date last accessed).

[17] de Boysson H, Daumas A, Vautier M et al Large-vessel involvement and aortic dilation in giant-cell arteritis. A multicenter study of 549 patients. Autoimmun Rev 2018;17:391–8. 29427822 10.1016/j.autrev.2017.11.029

[18] Smith R, Kuet KP, Akil M, Kilding R. Is mycophenolate mofetil effective in the treatment of large vessel vasculitis? Annals of the Rheumatic Diseases 2015;74:525.

[19] Hellmich B, Agueda A, Monti S et al 2018 Update of the EULAR recommendations for the management of large vessel vasculitis. Ann Rheum Dis 2020;79:19–30. 31270110 10.1136/annrheumdis-2019-215672

[20] Stone JH, Tuckwell K, Dimonaco S et al Trial of tocilizumab in giant-cell arteritis. N Engl J Med 2017;377:317–28. 28745999 10.1056/NEJMoa1613849

[21] Mahr AD, Jover JA, Spiera RF et al Adjunctive methotrexate for treatment of giant cell arteritis: an individual patient data meta-analysis. Arthritis Rheum 2007;56:2789–97. 17665429 10.1002/art.22754

